# Circulation of Rhinoviruses and/or Enteroviruses in Pediatric Patients With Acute Respiratory Illness Before and During the COVID-19 Pandemic in the US

**DOI:** 10.1001/jamanetworkopen.2022.54909

**Published:** 2023-02-07

**Authors:** Danielle A. Rankin, Andrew J. Spieker, Ariana Perez, Anna L. Stahl, Herdi K. Rahman, Laura S. Stewart, Jennifer E. Schuster, Joana Y. Lively, Zaid Haddadin, Varvara Probst, Marian G. Michaels, John V. Williams, Julie A. Boom, Leila C. Sahni, Mary A. Staat, Elizabeth P. Schlaudecker, Monica M. McNeal, Christopher J. Harrison, Geoffrey A. Weinberg, Peter G. Szilagyi, Janet A. Englund, Eileen J. Klein, Susan I. Gerber, Meredith McMorrow, Brian Rha, James D. Chappell, Rangaraj Selvarangan, Claire M. Midgley, Natasha B. Halasa, and the NVSN Network Investigators

**Affiliations:** 1 Department of Pediatrics, Vanderbilt University Medical Center, Nashville, Tennessee; 2 Vanderbilt Epidemiology PhD Program, Vanderbilt University School of Medicine, Nashville, Tennessee; 3 Department of Biostatistics, Vanderbilt University Medical Center, Nashville, Tennessee; 4 Division of Viral Diseases, National Center for Immunization and Respiratory Diseases, Centers for Disease Control and Prevention, Atlanta, Georgia; 5 General Dynamics Information Technology Inc, Falls Church, Virginia; 6 Division of Pediatric Infectious Diseases, Children’s Mercy Kansas City, Kansas City, Missouri; 7 Department of Pediatrics, University of Pittsburgh School of Medicine, University of Pittsburgh Medical Center Children’s Hospital of Pittsburgh, Pittsburgh, Pennsylvania; 8 Department of Pediatrics, Baylor College of Medicine, Houston, Texas; 9 Texas Children’s Hospital, Houston; 10 Division of Infectious Diseases, Department of Pediatrics, University of Cincinnati College of Medicine, Cincinnati Children’s Hospital Medical Center, Cincinnati, Ohio; 11 Department of Pediatrics, University of Rochester School of Medicine and Dentistry, Rochester, New York; 12 Department of Pediatrics, UCLA (University of California, Los Angeles) Mattel Children’s Hospital, UCLA, Los Angeles; 13 Seattle Children’s Hospital, Department of Pediatrics, University of Washington School of Medicine, Seattle; 14 Department of Pathology and Laboratory Medicine, Children’s Mercy Kansas City, Kansas City, Missouri

## Abstract

**Importance:**

Rhinoviruses and/or enteroviruses, which continued to circulate during the COVID-19 pandemic, are commonly detected in pediatric patients with acute respiratory illness (ARI). Yet detailed characterization of rhinovirus and/or enterovirus detection over time is limited, especially by age group and health care setting.

**Objective:**

To quantify and characterize rhinovirus and/or enterovirus detection before and during the COVID-19 pandemic among children and adolescents seeking medical care for ARI at emergency departments (EDs) or hospitals.

**Design, Setting, and Participants:**

This cross-sectional study used data from the New Vaccine Surveillance Network (NVSN), a multicenter, active, prospective surveillance platform, for pediatric patients who sought medical care for fever and/or respiratory symptoms at 7 EDs or hospitals within NVSN across the US between December 2016 and February 2021. Persons younger than 18 years were enrolled in NVSN, and respiratory specimens were collected and tested for multiple viruses.

**Main Outcomes and Measures:**

Proportion of patients in whom rhinovirus and/or enterovirus, or another virus, was detected by calendar month and by prepandemic (December 1, 2016, to March 11, 2020) or pandemic (March 12, 2020, to February 28, 2021) periods. Month-specific adjusted odds ratios (aORs) for rhinovirus and/or enterovirus–positive test results (among all tested) by setting (ED or inpatient) and age group (<2, 2-4, or 5-17 years) were calculated, comparing each month during the pandemic to equivalent months of previous years.

**Results:**

Of the 38 198 children and adolescents who were enrolled and tested, 11 303 (29.6%; mean [SD] age, 2.8 [3.7] years; 6733 boys [59.6%]) had rhinovirus and/or enterovirus–positive test results. In prepandemic and pandemic periods, rhinoviruses and/or enteroviruses were detected in 29.4% (9795 of 33 317) and 30.9% (1508 of 4881) of all patients who were enrolled and tested and in 42.2% (9795 of 23 236) and 73.0% (1508 of 2066) of those with test positivity for any virus, respectively. Rhinoviruses and/or enteroviruses were the most frequently detected viruses in both periods and all age groups in the ED and inpatient setting. From April to September 2020 (pandemic period), rhinoviruses and/or enteroviruses were detectable at similar or lower odds than in prepandemic years, with aORs ranging from 0.08 (95% CI, 0.04-0.19) to 0.76 (95% CI, 0.55-1.05) in the ED and 0.04 (95% CI, 0.01-0.11) to 0.71 (95% CI, 0.47-1.07) in the inpatient setting. However, unlike some other viruses, rhinoviruses and/or enteroviruses soon returned to prepandemic levels and from October 2020 to February 2021 were detected at similar or higher odds than in prepandemic months in both settings, with aORs ranging from 1.47 (95% CI, 1.12-1.93) to 3.01 (95% CI, 2.30-3.94) in the ED and 1.36 (95% CI, 1.03-1.79) to 2.44 (95% CI, 1.78-3.34) in the inpatient setting, and in all age groups. Compared with prepandemic years, during the pandemic, rhinoviruses and/or enteroviruses were detected in patients who were slightly older, although most (74.5% [1124 of 1508]) were younger than 5 years.

**Conclusions and Relevance:**

Results of this study show that rhinoviruses and/or enteroviruses persisted and were the most common respiratory virus group detected across all pediatric age groups and in both ED and inpatient settings. Rhinoviruses and/or enteroviruses remain a leading factor in ARI health care burden, and active ARI surveillance in children and adolescents remains critical for defining the health care burden of respiratory viruses.

## Key Points

**Question:**

How commonly were rhinoviruses and/or enteroviruses detected in children and adolescents seeking medical attention before and during the first year of the COVID-19 pandemic, and did detections differ by age group or health care setting?

**Findings:**

In this cross-sectional study of 38 198 pediatric patients who underwent respiratory specimen testing, rhinoviruses and/or enteroviruses were the most frequently detected respiratory viruses in the emergency department and hospital setting before and during the pandemic.

**Meaning:**

Findings of this study suggest that rhinoviruses and/or enteroviruses remain a major factor in acute respiratory illness in pediatric patients, including those hospitalized; active surveillance is critical for defining the health care burden of respiratory viruses in this patient population.

## Teaser

This cross-sectional study describes the detection of rhinoviruses and/or enteroviruses by pediatric age group and health care setting.

## Introduction

Sharp declines in circulation of enveloped respiratory viruses have been described globally,^1,2,3,4,5,6,7,8,9^ including in the US, in temporal association with the COVID-19 pandemic and nonpharmaceutical interventions (eg, masking, social distancing, and handwashing).^10,11,12,13^ Despite these interventions, rhinoviruses (ie, nonenveloped viruses that are sometimes combined with enteroviruses and reported as rhinoviruses and/or enteroviruses because of molecular cross-reactivity between the 2 groups) were not affected as substantially,^1,2,3,4,5,6,7,8,10,11,12^ although enterovirus D68 circulation was lower than anticipated in the fall of 2020.^14^ Continued circulation of rhinoviruses (or rhinovirus and/or enterovirus) has been attributed to viral stability; the possible role of asymptomatic transmission; and/or the relative contribution of different transmission mechanisms, such aerosol, droplet, or contact.^1,2,3,4,5,6,7,8,10,11,12^ However, these previous reports describing rhinovirus (or rhinovirus and/or enterovirus) circulation during the pandemic were restricted to the fall of 2020,^4,5,7,8,9^ were among heterogeneous populations (unspecific to age group and/or health care setting),^4,5,6,7,9,10,11,12^ or had limited clinical descriptions.^1,2,3,4,5,6,7,8,9,10,11,12^ Therefore, in this cross-sectional study, we aimed to quantify and characterize rhinovirus and/or enterovirus detection across multiple years before and during the COVID-19 pandemic among children and adolescents seeking medical care for acute respiratory illness (ARI) at emergency departments (EDs) or hospitals within the New Vaccine Surveillance Network (NVSN) in the US.

New Vaccine Surveillance Network is a multicenter surveillance platform funded by the Centers for Disease Control and Prevention. Since 2016, NVSN has conducted active, prospective year-round ARI surveillance in 7 US cities of children and adolescents with fever and/or at least 1 respiratory symptom presenting to the ED or requiring hospitalization. A previous NVSN investigation demonstrated that circulation of non–SARS-CoV-2 enveloped respiratory viruses (eg, influenza and respiratory syncytial virus [RSV]) sharply declined after the implementation of nonpharmaceutical interventions for SARS-CoV-2 in all 7 US cities, which differed from prior respiratory seasons (October-April 2017-2020).^13^ Unlike RSV and influenza circulation, which prior to emergence of SARS-CoV-2 had fall or winter peaks, rhinovirus circulation typically occurs year-round, with circulation peaks mostly in the spring and early fall. The geographic breadth, multiple years of surveillance data, and large study population positioned NVSN to define patterns of rhinovirus and/or enterovirus–associated, medically attended ARI and the associated clinical outcome before and during the COVID-19 pandemic.

## Methods

### Study Design and Population

This cross-sectional study involved persons younger than 18 years who were enrolled in NVSN from December 2016 through February 2021.^13,15,16^ The institutional review boards at each of the 7 surveillance sites (Children’s Hospital of Pittsburgh of the University of Pittsburgh Medical Center, Pittsburgh, Pennsylvania; Children’s Mercy Hospital and Clinics, Kansas City, Missouri; Cincinnati Children’s Hospital Medical Center, Cincinnati, Ohio; Seattle Children’s Hospital, Seattle, Washington; Texas Children’s Hospital, Houston, Texas; University of Rochester, Rochester, New York; and Vanderbilt University Medical Center, Nashville, Tennessee [eTables 1-3 in Supplement 1]) and the Centers for Disease Control and Prevention reviewed and approved this study.^17^ All parents or guardians provided written informed consent and assent when applicable. We followed the Strengthening the Reporting of Observational Studies in Epidemiology (STROBE) reporting guideline.

Eligible children and adolescents resided within each site’s catchment or surveillance area and presented to an ED or were hospitalized within 48 hours of enrollment. Respiratory specimens (eg, midturbinate nasal, oropharyngeal, and/or tracheal aspirates) were collected and tested for respiratory viruses. Comprehensive descriptions of the study design and eligibility criteria are provided in the eMethods in Supplement 1.

### Statistical Analysis

Virus-specific proportions among all children and adolescents with respiratory specimen testing and among those with a virus-positive test result were evaluated by calendar months for nonenveloped respiratory viruses, SARS-CoV-2, and other respiratory enveloped viruses (eMethods in Supplement 1). Sociodemographic characteristics (including race and ethnicity reported by NVSN sites with these categories: Hispanic; non-Hispanic Black; non-Hispanic White; and other [American Indian or Alaska Native, Asian, and Native Hawaiian or other Pacific Islander]) and clinical characteristics of pediatric patients in whom rhinovirus and/or enterovirus was detected were summarized by period (prepandemic period, defined as December 1, 2016, to March 11, 2020, or pandemic period, defined as March 12, 2020, to February 28, 2021) using frequency (%) for categorical variables and mean (SD) for continuous variables. Comparisons were conducted using either an unpaired, 2-tailed *t* test with unequal variances or Pearson χ^2^ test as appropriate.

Adjustment variables were selected a priori. We used logistic regression to estimate month-specific adjusted odds ratios (aORs) and 95% CIs for the proportion of respiratory specimens with rhinovirus and/or enterovirus–positive test results each month in the pandemic period compared with the corresponding calendar months in the prepandemic period (eg, April 2020 vs April 2017, 2018, and 2019) and were further stratified by setting (ED or inpatient) and age group (<2, 2-4, or 5-17 years).^14,15,16^ We adjusted for age (continuous), sex (male or female), insurance type (private, public, or self-pay), and enrollment site. Generalized estimating equations with a working independence correlation structure were used to account for within-patient correlation (ie, patients contributing more than 1 observation [ED visit or hospitalization] during the study period). To address missing data for insurance type (total number of data missing, 134), multiple imputation was performed using chained equations.

Statistical significance was determined based on a level of 2-tailed α = .05. Statistical analyses were performed in R, version 3.6.1 (R Foundation for Statistical Computing).

## Results

From December 2016 to February 2021, 38 950 (55.5%) of 70 162 eligible children and adolescents were enrolled in NVSN, among whom 38 198 (98.1%) had respiratory specimen testing. Of these pediatric patients, 11 303 (29.6%) had rhinovirus and/or enterovirus–positive test results (eFigure 1 in Supplement 1). The patients included 4570 girls (40.4%) and 6733 boys (59.6%), with a mean (SD) age of 2.8 (3.7) years (Table 1).

**Table 1.** Sociodemographic Characteristics of Children and Adolescents With Rhinovirus and/or Enterovirus–Positive Test Results in the Prepandemic and Pandemic Periods

Characteristic	ED			Inpatient		
	No. (%)		*P* value^a^	No. (%)		*P* value^a^
	Prepandemic period (n = 4621)	Pandemic period (n = 841)		Prepandemic period (n = 5174)	Pandemic period (n = 667)	
Age						
Mean (SD), y	2.5 (3.3)	3.0 (3.9)	<.001	3.0 (3.8)	3.7 (4.4)	<.001
Median (IQR), y	1.0 (0.0-3.0)	2.0 (0.0-4.0)		1.0 (0.0-4.0)	2.0 (1.0-6.0)	
<2 y	2544 (55.1)	409 (48.6)	<.001	2688 (52.0)	305 (45.7)	<.001
2-4 y	1353 (29.3)	247 (29.4)		1226 (23.7)	163 (24.4)	
5-17 y^b^	724 (15.7)	185 (22.0)		1260 (24.4)	199 (29.8)	
Sex						
Female	1899 (41.1)	350 (41.6)	.81	2038 (39.4)	283 (42.4)	.14
Male	2722 (58.9)	491 (58.4)		3136 (60.6)	384 (57.6)	
Race and ethnicity						
Hispanic	1136 (24.6)	181 (21.5)	<.001	1158 (22.4)	138 (20.7)	<.001
Non-Hispanic Black	2009 (43.5)	339 (40.3)		1391 (26.9)	192 (28.8)	
Non-Hispanic White	1040 (22.5)	242 (28.8)		1999 (38.6)	257 (38.5)	
Other non-Hispanic^c^	421 (9.1)	70 (8.3)		606 (11.7)	69 (10.3)	
Unknown	15 (0.3)	9 (1.1)		20 (0.4)	11 (1.6)	
Insurance type						
Public	3457 (74.8)	622 (74.0)	.46	3278 (63.4)	426 (63.9)	.13
Private	792 (17.1)	161 (19.1)		1536 (29.7)	209 (31.3)	
Both	40 (0.9)	8 (1.0)		64 (1.2)	3 (0.4)	
None or self-pay	270 (5.8)	39 (4.6)		243 (4.7)	21 (3.1)	
Unknown	62 (1.3)	11 (1.3)		53 (1.0)	8 (1.2)	
Social exposures						
Daycare or school attendance^d^	2363 (51.1)	318 (37.8)	<.001	2561 (49.5)	218 (32.7)	<.001
Smoke exposure	1322 (28.6)	221 (26.3)	.18	1454 (28.1)	190 (28.5)	.87
e-Cigarette exposure	185 (4.0)	36 (4.3)	.78	255 (4.9)	39 (5.8)	.35
Surveillance sites						
Nashville, Tennessee	539 (11.7)	25 (3.0)	<.001	398 (7.7)	76 (11.4)	<.001
Rochester, New York	537 (11.6)	66 (7.8)		642 (12.4)	39 (5.8)	
Cincinnati, Ohio	399 (8.6)	173 (20.6)		531 (10.3)	81 (12.1)	
Seattle, Washington	614 (13.3)	94 (11.2)		569 (11.0)	48 (7.2)	
Houston, Texas	457 (9.9)	73 (8.7)		935 (18.1)	95 (14.2)	
Kansas City, Kansas	1227 (26.6)	270 (32.1)		677 (13.1)	103 (15.4)	
Pittsburgh, Pennsylvania	848 (18.4)	140 (16.6)		1422 (27.5)	225 (33.7)	
Respiratory specimen results						
Any viral codetection	1149 (24.9)	71 (8.4)	<.001	1000 (19.3)	36 (5.4)	<.001
Adenovirus	419 (9.1)	44 (5.2)	<.001	275 (5.3)	18 (2.7)	.005
Other viruses^e^	815 (17.6)	13 (1.5)	<.001	799 (15.4)	5 (0.7)	<.001
SARS-CoV-2	NA	17 (2.0)	NA	NA	14 (2.1)	NA

Abbreviations: ED, emergency department; NA, not applicable.

^a^ *P* values comparing prepandemic and pandemic periods were calculated using an unpaired, 2-tailed *t* test with unequal variances for continuous variables and Pearson χ^2^ test for categorical variables, with α<.05.

^b^ Inpatient enrollment was year-round at all 7 surveillance sites and included patients younger than 18 years. Meanwhile, ED enrollment at 3 surveillance sites was restricted to patients younger than 5 years for some months (eTable 1 in Supplement 1).

^c^ Other non-Hispanic race and ethnicity included American Indian or Alaska Native, Asian, and Native Hawaiian or other Pacific Islander.

^d^ History of daycare or school attendance.

^e^ Other viruses included parainfluenza (types 1-4), influenza, human metapneumovirus, and respiratory syncytial virus.

Beginning December 2016, the number of eligible patients fluctuated over time, with peaks in winter months. In March 2020, both the number of eligible and the number of enrolled patients sharply decreased, which was sustained through the remainder of the study period (eFigure 2A in Supplement 1). Despite the decrease of eligible patients, the percentage of those enrolled remained constant (mean of 55.0%) throughout the pandemic period.

### Detection of Respiratory Viruses

**Figure 1.**
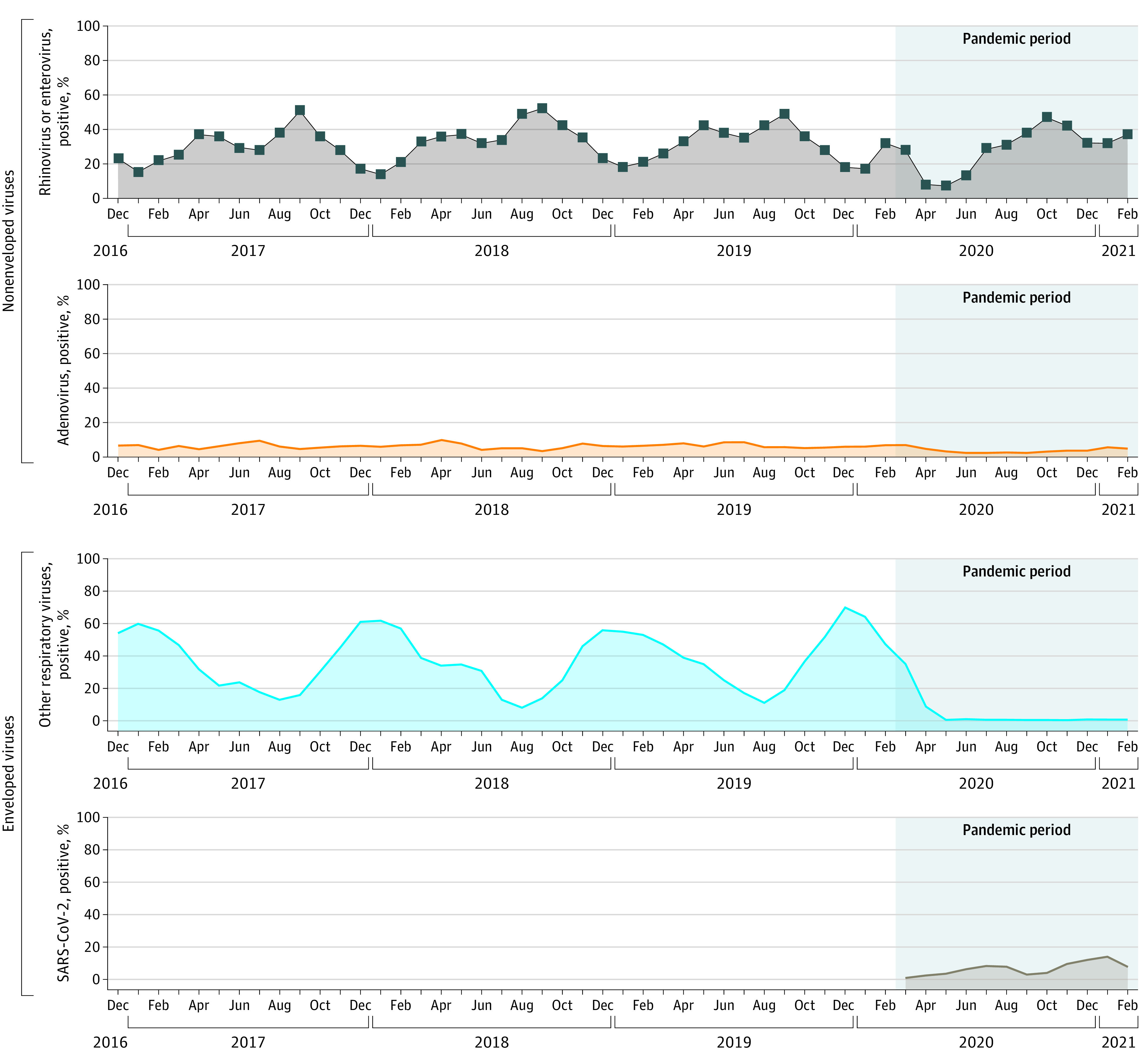
Virus Circulation Among Children and Adolescents With Respiratory Virus Testing, From 2016 to 2021 Other respiratory viruses included influenza, parainfluenza types 1 to 4, respiratory syncytial virus, and human metapneumovirus.

A total of 23 236 of 33 317 patients (69.7%) who were tested had at least 1 respiratory virus–positive result during the prepandemic period compared with 2066 of 4881 patients (42.3%) during the pandemic period. In the prepandemic and pandemic periods, respectively, rhinoviruses and/or enteroviruses were detected in 29.4% (9795 of 33 317) and 30.9% (1508 of 4881) of patients who were enrolled and tested (Figure 1) as well as in 42.2% (9795 of 23 236) and 73.0% (1508 of 2066) of patients with at least 1 respiratory virus–positive test result.

Rhinoviruses and/or enteroviruses were detected year-round, with peaks in late summer or early fall and spring. Soon after the start of the pandemic, the positivity rate for rhinoviruses and/or enteroviruses decreased but largely returned to almost prepandemic levels by the fall of 2020 (with a pandemic period mean viral test positivity rate of 37.0%) (Figure 1; eFigure 2B in Supplement 1). Similarly, adenovirus circulated year-round, with a moderate decline in March 2020, and this low level of circulation continued through February 2021 (with a pandemic period mean viral test positivity rate of 3.8%) (Figure 1). Meanwhile, enveloped viruses (RSV, influenza, human metapneumovirus, and parainfluenza types 1-4) were predominant during the winter months, but substantive decreases in circulation were observed early in the pandemic period, with negligible detection throughout the remainder of the pandemic period (Figure 1). The first SARS-CoV-2 detection in NVSN was in March 2020,^18^ and the mean SARS-CoV-2 positivity rate for the pandemic period was 6.6% among children and adolescents with respiratory specimen testing.

### Rhinovirus and/or Enterovirus Detection by Period, Age Group, and Setting

Rhinoviruses and/or enteroviruses were the most frequently detected virus group by period, age group, and health care setting. Among patients with any virus detected, rhinoviruses and/or enteroviruses were detected in 39.8% (5232 of 13 144) of those younger than 2 years, 43.2% (2579 of 5974) of those 2 to 4 years of age, and 48.2% (1984 of 4118) of those 5 to 17 years of age during the prepandemic period. Rhinoviruses and/or enteroviruses were detected in 72.0% (714 of 991) of patients younger than 2 years, 84.7% (410 of 484) of those 2 to 4 years of age, and 65.0% (384 of 591) of those 5 to 17 years of age during the pandemic period. Among patients in the ED with any virus detected, 40.0% (4621 of 11 561) in the prepandemic period and 72.6% (841 of 1159) in the pandemic period had rhinovirus and/or enterovirus–positive test results. Among pediatric inpatients with any virus detected, 44.3% (5174 of 11 675) had rhinovirus and/or enterovirus–positive test results during the prepandemic period compared with 73.5% (667 of 907) with positive test results during the pandemic period.

**Figure 2.**
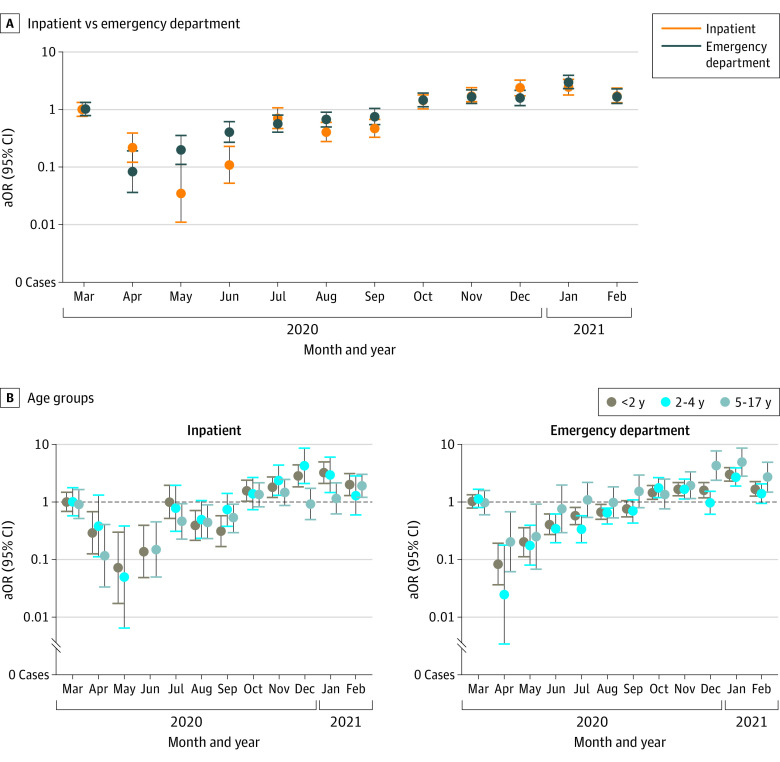
Adjusted Odds Ratios (aORs) of Rhinovirus and/or Enterovirus Detection in the Inpatient and Emergency Department Settings and Across Age Groups, From March 2020 to February 2021 vs From December 2016 to February 2020 Models were adjusted for age (continuous), sex, and insurance type (public, private, or self-pay), with fixed effects for surveillance sites. March 2020 was a transition month, which included patients in both the prepandemic and pandemic periods. The error bars represent 95% CIs.

The month-specific aORs and 95% CIs for the detection of rhinoviruses and/or enteroviruses during the pandemic period compared with equivalent months in the prepandemic years are shown by setting and age group in Figure 2 and eTables 4 and 5 in Supplement 1. From April to September 2020 in all age groups and in both the ED and inpatient settings, rhinoviruses and/or enteroviruses were detected at similar or lower odds compared with equivalent months in prepandemic years in both settings, with aORs ranging from 0.08 (95% CI, 0.04-0.19) to 0.76 (95% CI, 0.55-1.05) in the ED and 0.04 (95% CI, 0.01-0.11) to 0.71 (95% CI, 0.47-1.07) in the inpatient setting (Figure 2; eTables 4 and 5 in Supplement 1). However, similar or higher aORs were observed between October 2020 and February 2021 compared with corresponding months in the prepandemic years, ranging from 1.47 (95% CI, 1.12-1.93) to 3.01 (95% CI, 2.30-3.94) in the ED and 1.36 (95% CI, 1.03-1.79) to 2.44 (95% CI, 1.78-3.34) in the inpatient setting (eTable 4 in Supplement 1).

### Viral Codetection With Rhinovirus and/or Enterovirus

**Figure 3.**
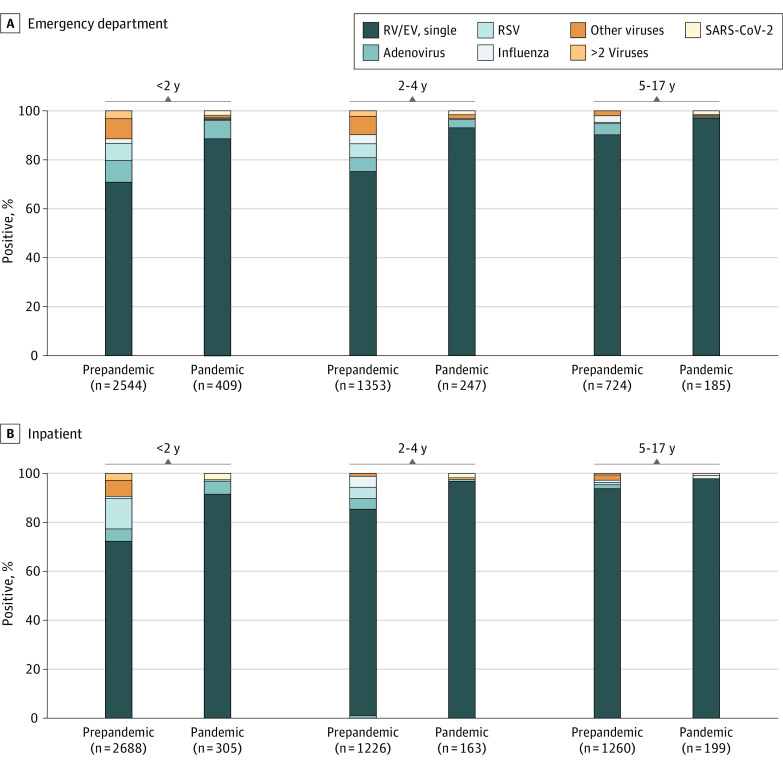
Rhinovirus and/or Enterovirus (RV/EV) Single Detection or Codetection With Other Respiratory Viruses in the Prepandemic vs Pandemic Periods by Age Groups Other respiratory viruses included influenza, parainfluenza types 1 to 4, respiratory syncytial virus (RSV), and human metapneumovirus.

The frequency of virus codetection among patients with rhinovirus and/or enterovirus–positive test results was 21.9% (24.9% [1149] in the ED vs 19.3% [1000] in the inpatient setting; *P* < .001) in the prepandemic period compared with 7.1% (8.4% [71] in the ED vs 5.4% [36] in the inpatient setting; *P* < .001) during the pandemic period (Table 1). Adenovirus was the most common virus codetected among patients in the ED during both periods and among inpatients during the pandemic (Table 1). In approximately 2% of children in both settings, rhinovirus and/or enterovirus was codetected with SARS-CoV-2. Overall, the age-specific proportions of rhinovirus and/or enterovirus codetection with any other respiratory viruses were 28.4% (1487 of 5232) in the younger-than-2-years age group, 19.8% (511 of 2579) in the 2-to-4-years age group, and 7.6% (151 of 1984) in the 5-to-17-years age group during the prepandemic period (Figure 3). In the pandemic period, these overall proportions decreased to 10.4% (74 of 714) in the younger-than-2-years age group, 4.5% (22 of 488) in the 2-to-4-years age group, and 2.9% (11 of 384) in the 5-to-17-years age group, which were consistent with the reduced circulation of other viruses.

### Demographic and Clinical Characteristics of Patients With Rhinovirus and/or Enterovirus in ED and Inpatient Settings

Sociodemographic characteristics of children and adolescents in whom rhinovirus and/or enterovirus was detected are displayed in Table 1 (stratified by period and health care setting). Overall, most patients with rhinovirus and/or enterovirus were younger than 5 years (79.0% [8935]), were boys (59.6% [6733]), or had public health insurance (68.9% [7783]). Compared with the prepandemic period, those with a rhinovirus and/or enterovirus detected during the pandemic in both ED and inpatient settings were older (mean age difference of approximately 6 months; 74.5% [1124 of 1508] were younger than 5 years) and attended daycare or school less frequently (Table 1). In the ED, the proportion of patients with rhinovirus and/or enterovirus–positive test results who were of non-Hispanic White race and ethnicity was greater during the pandemic vs the prepandemic period (28.8% [242] vs 22.5% [1040]). In the inpatient setting, however, the race and ethnicity distribution was largely similar across both time periods, although a slightly higher proportion of inpatients during the pandemic period were of non-Hispanic Black race and ethnicity (28.8% [192] vs 26.9% [1391]) (Table 1). In addition, differences by surveillance site were observed.

During the prepandemic and pandemic periods, respectively, 47.1% (4621 of 9803) and 55.8% (841 of 1508) of patients with rhinovirus and/or enterovirus–positive test results were seen in the ED but not admitted. Among those with rhinovirus and/or enterovirus–positive test results in the ED, the proportion with at least 1 underlying medical condition was comparable between both periods (33.2% [1532] vs 31.3% [263]), although underlying respiratory conditions (including asthma) were more frequent in the pandemic period (Table 2). However, compared with the prepandemic period, patients in the pandemic period were less likely to present with wheezing (22.2% [187] vs 30.9% [1424]) and retractions (16.4% [138] vs 24.8% [1137]). Furthermore, the mean (SD) time to presentation from onset of illness symptoms was longer among patients with rhinovirus and/or enterovirus–positive test results during the pandemic period compared with the prepandemic period (2.6 [2.2] vs 1.2 [2.1] days) (Table 2). The most frequent ARI-related discharge diagnosis was acute upper respiratory infection in both periods. In the pandemic period, discharge diagnoses that were consistent with respiratory viruses were less frequent than in the prepandemic period (eg, bronchiolitis in the ED: 44 [5.2%] vs 415 [9.0%]).

**Table 2.** Clinical Characteristics of Children and Adolescents With Rhinovirus and/or Enterovirus–Positive Test Results in the Prepandemic and Pandemic Periods

Characteristic	ED			Inpatient		
	No. (%)		*P* value^a^	No. (%)		*P* value^a^
	Prepandemic period (n = 4621)	Pandemic period (n = 841)		Prepandemic period (n = 5174)	Pandemic period (n = 667)	
Underlying medical conditions^b^						
≥1 Underlying medical condition	1532 (33.2)	263 (31.3)	.30	2902 (56.1)	383 (57.4)	.54
Respiratory	642 (13.9)	149 (17.7)	.004	1463 (28.3)	257 (38.5)	<.001
Asthma or RAD only	554 (12.0)	125 (14.9)	.02	1187 (22.9)	208 (31.2)	<.001
Cardiovascular	81 (1.8)	25 (3.0)	.03	250 (4.8)	44 (6.6)	.06
Neurological or neuromuscular	144 (3.1)	56 (6.7)	<.001	419 (8.1)	93 (13.9)	<.001
Oncologic or immunosuppressive	428 (9.3)	128 (15.2)	<.001	936 (18.1)	203 (30.4)	<.001
Kidney	13 (0.3)	6 (0.7)	.10	36 (0.7)	4 (0.6)	.97
Gastrointestinal or hepatic	124 (2.7)	25 (3.0)	.72	356 (6.9)	68 (10.2)	.002
Endocrine	10 (0.2)	5 (0.6)	.12	54 (1.0)	15 (2.2)	.01
Other	368 (8.0)	12 (1.4)	<.001	576 (11.1)	23 (3.4)	<.001
Prematurity: <37 wk^c^	148 (3.2)	69 (8.2)	<.001	301 (5.8)	101 (15.1)	<.001
Physical assessment						
Wheezing	1424 (30.9)^d^	187 (22.2)	<.001	3359 (65.2)^e^	420 (63.0)	.26
Retractions	1137 (24.8)^f^	138 (16.4)^g^	<.001	3589 (70.0)^h^	426 (64.4)^i^	.004
Illness duration, d						
Mean (SD)	1.2 (2.1)	2.6 (2.2)	<.001	1.5 (2.3)	3.0 (2.3)	<.001
Median (IQR)	0 (0.0-2.0)	2.0 (1.0-3.0)		0 (0.0-2.0)	2.0 (1.0-4.0)	
Primary discharge diagnosis						
Acute upper respiratory infection	843 (18.2)	156 (18.5)	.87	189 (3.7)	25 (3.7)	.99
Acute bronchiolitis	415 (9.0)	44 (5.2)	<.001	1048 (20.3)	105 (15.7)	.007
Asthma or RAD	668 (14.5)	68 (8.1)	<.001	927 (17.9)	133 (19.9)	.22
Fever, unspecified	319 (6.9)	71 (8.4)	.13	93 (1.8)	11 (1.6)	.91
Viral infection, unspecified	207 (4.5)	81 (9.6)	<.001	51 (1.0)	10 (1.5)	.31
Pneumonia	86 (1.9)	6 (0.7)	.03	262 (5.1)	24 (3.6)	.12
Acute respiratory failure with hypoxia	0	0	NA	342 (6.6)	30 (4.5)	.04
Croup	181 (3.9)	19 (2.3)	.02	114 (2.2)	7 (1.0)	.07
Primary admission diagnosis						
Asthma or RAD	NA	NA	NA	969 (18.7)	111 (16.6)	.21
Acute bronchiolitis	NA	NA	NA	861 (16.6)	81 (12.1)	.004
Fever, unspecified	NA	NA	NA	277 (5.4)	64 (9.6)	<.001
Pneumonia	NA	NA	NA	200 (3.9)	14 (2.1)	.03
Cough	NA	NA	NA	186 (3.6)	28 (4.2)	.50
Periodic breathing	NA	NA	NA	167 (3.2)	44 (6.6)	<.001

Abbreviations: ED, emergency department; NA, not applicable; RAD, reactive airway disease.

^a^ *P* values comparing prepandemic and pandemic periods were calculated using an unpaired, 2-tailed *t* test with unequal variances for continuous variables and Pearson χ^2^ test for categorical variables, with α<.05.

^b^ Underlying medical conditions were based on medical record review.

^c^ History of prematurity was reported for patients younger than 2 years.

^d^ n = 4603.

^e^ n = 5148.

^f^ n = 4578.

^g^ n = 839.

^h^ n = 5129.

^i^ n = 661.

Among patients with rhinovirus and/or enterovirus–positive test results, a greater proportion required inpatient care during the prepandemic period (52.8% [5174 of 9795]) than the pandemic period (44.2% [667 of 1508]). Among inpatients with rhinovirus and/or enterovirus–positive test results, the proportion with at least 1 underlying medical condition was comparable between both periods (56.1% [2902] vs 57.4% [383]), although respiratory conditions (including asthma) were more prevalent in the pandemic than the prepandemic period (Table 2). In the inpatient setting, no differences in the frequency of patients identified with wheezing during their physical assessment were found between the prepandemic and pandemic periods. Patients with rhinovirus and/or enterovirus–positive test results who were enrolled in NVSN during the pandemic period had a longer mean (SD) time from symptom onset to presentation compared with those enrolled during the prepandemic period (3.0 [2.3] vs 1.5 [2.3] days). In both periods, asthma or reactive airway disease was among the most common admission and discharge diagnoses. Bronchiolitis admission and discharge diagnoses decreased in the pandemic period.

## Discussion

Rhinoviruses and/or enteroviruses were the most common viruses detected in children and adolescents with medically attended ARI during the 3 years prior to the COVID-19 pandemic and during the first year of the pandemic. Rhinoviruses and/or enteroviruses reflected just under half of all viral detections before the pandemic and nearly three-quarters during the pandemic. This high prevalence was observed in both the ED and inpatient settings and in all age groups, highlighting the continued importance of this virus group in the pediatric health care burden of ARI. Although rhinovirus and/or enterovirus detection decreased at the beginning of the pandemic period, the proportion of patients with rhinovirus and/or enterovirus had increased to prepandemic or higher levels by October 2020. Adenovirus, also a nonenveloped virus, continued to circulate throughout the pandemic period, although with lower relative frequency than the prepandemic period. In contrast, circulation of enveloped respiratory viruses (eg, RSV and influenza) sharply declined in the beginning of the pandemic period, and detection remained minimal throughout the study period. Consistent with this finding, viral codetection (among patients in whom rhinoviruses and/or enteroviruses were detected) also decreased during the pandemic. Despite virus codetection being more common in younger patients, rhinoviruses and/or enteroviruses were most often detected as a single virus in all age groups, especially in the inpatient setting, further highlighting the role of rhinoviruses and/or enteroviruses in severe ARI.

We believe that findings from the active, prospective ARI surveillance platform of NVSN strengthen previous evidence from the US and other countries that rhinoviruses and/or enteroviruses, unlike most other viruses, persisted during the early stages of the pandemic. In addition, enterovirus D68 (a single enterovirus type) also circulated in the US at low levels during the fall of 2020.^14^ Since the end of the study period (February 2021), reemergence of enveloped respiratory viruses has been reported in the US, sometimes out of the prepandemic expected seasons.^19,20^ Persistence of rhinoviruses and/or enteroviruses, and to some degree adenoviruses, when other respiratory viruses were substantially curtailed suggests that nonenveloped viruses were less affected by the various combinations of nonpharmaceutical interventions implemented (eg, masking, hand hygiene, use of surface disinfectants, or school or daycare closures). The reasons for these differences by virus are unclear, but a multitude of virologic, environmental, and/or behavioral factors may have contributed, including the stability of these nonenveloped viruses on surfaces and/or most prominent transmission routes.^21,22,23^ Further research is needed to better understand the continued circulation of nonenveloped compared with enveloped respiratory viruses during the early period of the pandemic.

Among the rhinoviruses and/or enteroviruses detected in this study, the majority were single detections (ie, no viral codetection), especially in inpatients or older pediatric patients, suggesting that rhinovirus infections play a role in severe ARI. The findings also reiterate the annual importance of rhinovirus and/or enterovirus in young patients, including those younger than 2 years,^24^ in whom other viruses, such as RSV, are sometimes considered more prominent based on high circulation in winter months.^16,25^ Before the pandemic, among pediatric patients with a rhinovirus and/or enterovirus detection, RSV and adenovirus were the most common codetected viruses in the inpatient and ED settings, respectively, a finding that is consistent with reports in other prepandemic studies.^3,26,27^ However, consistent with the decline of many other viruses beginning March 2020, we observed a decrease in viral codetection during the pandemic period, with adenovirus becoming the most common virus codetected in both settings, followed by SARS-CoV-2. The observed decrease of rhinovirus and RSV codetection during the pandemic, especially in the inpatient setting, may account for the lower frequency of wheezing on physical examination and for clinical diagnoses of bronchiolitis. Additional research is needed to ascertain whether these findings on changes to discharge diagnoses are similar in other pediatric health care settings.

We found that the characteristics of children and adolescents with rhinovirus and/or enterovirus–positive test results were largely similar before and during the pandemic, although with some differences identified that may have reflected risk of exposure, risk of severe illness, or access to care during the pandemic. Patients in both settings were slightly older during the pandemic by a mean difference of approximately 6 months, which may have reflected increased risk of infection or severe illness in older pediatric patients after lower levels of rhinovirus and/or enterovirus exposure during the early pandemic period. We observed an increase in the mean time from onset of symptoms to seeking care, which is consistent with findings in other studies that have noted reluctance to seek care during the pandemic.^28,29^ However, we also noted that patients seen in the ED during the pandemic were more likely to be of non-Hispanic White race and ethnicity, which may suggest differences by race and ethnicity in reluctance to seek care or in access to care. The number of patients with underlying conditions were comparable between study periods; overall, one-third of patients with a rhinovirus and/or enterovirus detection in the ED and more than half of inpatients reported at least 1 underlying medical condition. Respiratory conditions (including asthma) were the most reported underlying conditions in both settings throughout the study period. These findings are congruent with findings that rhinoviruses are associated with asthma exacerbations in children and adolescents, requiring an escalation of care, such as hospitalization.^24,30,31^

### Strengths and Limitations

Key strengths of this study include the use of a multicenter, active, prospective surveillance platform with systematic enrollment, interview, and molecular testing conducted over multiple years of children and adolescents from both the inpatient and ED settings. However, the findings should be considered in the context of the study limitations.

First, changes in health care–seeking behaviors attributable to the COVID-19 pandemic may explain the decline in health care visits observed in the study population, especially during community closures (ie, April-May 2020). Although the absolute numbers of pediatric patients who were eligible and enrolled decreased during the pandemic period, the proportions of patients in whom rhinovirus and/or enterovirus was detected were consistent with the prepandemic period. Thus, we believe the changes in health care behaviors had minimal implications for the overall observation of rhinovirus and/or enterovirus circulation, especially when a patient needed escalation of care, including hospitalization. Second, while the study included 7 geographically dispersed locations in the US, some results may not be generalizable to the rhinovirus and/or enterovirus burden nationwide. Third, reverse transcriptase–polymerase chain reaction cannot distinguish rhinoviruses from enteroviruses. Additional typing by molecular sequencing is needed to discriminate rhinovirus and enterovirus species- and type-specific circulation patterns during the pandemic period. Fourth, the study did not include targets for seasonal coronaviruses (NL63, 229E, OC43, and HKU1), which may have underestimated the prevalence of other respiratory virus detection. Finally, some NVSN sites restricted ED enrollment to patients younger than 5 years for parts of the study period, meaning that we likely underestimated the ED burden of rhinoviruses and/or enteroviruses in those aged 5 to 17 years.

## Conclusions

For the first year of the COVID-19 pandemic as other respiratory viruses exhibited reduced circulation, rhinoviruses and/or enteroviruses quickly reemerged and remained a leading factor in ARI, accounting for approximately three-quarters of viral detections in pediatric health care visits. Active, systematic ARI surveillance in children and adolescents remains critical for defining the health care burden of respiratory viruses. Specifically, active ARI surveillance aids in identifying seasonality changes, risk factors for severe illness, and the implications of viral codetection, especially as SARS-CoV-2 transmission continues and the implementation of community interventions evolves.

## Notes

Supplement 1. **eMethods.** New Vaccine Surveillance Network Enrollment Eligibility Criteria and Research Surveillance Specimen Laboratory Testing Methods **eTable 1.** Age Group-Specific Enrollment by Site and Setting, New Vaccine Surveillance Network, 2016-21 **eTable 2.** Absolute Number of Rhinovirus/Enterovirus Detections and Total of Children Who Received Respiratory Viral Testing From Enrollments in the Emergency Department by Surveillance Site and Age-Groups, New Vaccine Surveillance Network, 2016-2021 **eTable 3.** Number of Children With Rhinovirus- and/or Enterovirus ARI Study Specimens, by RT-PCR Test Type and Time Period **eFigure 1.** Consort Diagram of Enrolled and Eligible Children, New Vaccine Surveillance Network, United States, November 2016–February 2021 **eFigure 2.** Frequency of Children Eligible and Enrolled (A) and the Absolute Number of Children With Rhinovirus/Enterovirus Detected From Research Respiratory Specimen (B), New Vaccine Surveillance Network, United States, December 2016–February 2021 **eTable 4.** Adjusted Odds Ratios of Rhinovirus/Enterovirus Test Positivity in the Inpatient and Emergency Department Settings From March 2020 to February 2021 Compared to the Same Month in Prior Years (2016-2020), United States **eTable 5.** Adjusted Odds Ratios of Rhinovirus/Enterovirus Test Positivity in the Inpatient and Emergency Department Settings From March 2020 to February 2021 Compared to the Same Month in Prior Years (2016-2020), United States, by Age Group

Supplement 2.
Nonauthor Collaborators

Supplement 3.
Data Sharing Statement
