# Supplementary material for: Circulation of Rhinoviruses and/or Enteroviruses in Pediatric Patients With Acute Respiratory Illness Before and During the COVID-19 Pandemic in the US
Source: JAMA Netw Open. 2023 Feb 7;6(2):e2254909. doi: 10.1001/jamanetworkopen.2022.54909 (PMC10408278; doi:10.1001/jamanetworkopen.2022.54909)
Supplement: Supplement 1. — eMethods. New Vaccine Surveillance Network Enrollment Eligibility Criteria and Research Surveillance Specimen Laboratory Testing Methods eTable 1. Age Group-Specific Enrollment by Site and Setting, New Vaccine Surveillance Network, 2016-21 eTable 2. Absolute Number of Rhinovirus/Enterovirus Detections and Total of Children Who Received Respiratory Viral Testing From Enrollments in the Emergency Department by Surveillance Site and Age-Groups, New Vaccine Surveillance Network, 2016-2021 eTable 3. Number of Children With Rhinovirus- and/or Enterovirus ARI Study Specimens, by RT-PCR Test Type and Time Period eFigure 1. Consort Diagram of Enrolled and Eligible Children, New Vaccine Surveillance Network, United States, November 2016–February 2021 eFigure 2. Frequency of Children Eligible and Enrolled (A) and the Absolute Number of Children With Rhinovirus/Enterovirus Detected From Research Respiratory Specimen (B), New Vaccine Surveillance Network, United States, December 2016–February 2021 eTable 4. Adjusted Odds Ratios of Rhinovirus/Enterovirus Test Positivity in the Inpatient and Emergency Department Settings From March 2020 to February 2021 Compared to the Same Month in Prior Years (2016-2020), United States eTable 5. Adjusted Odds Ratios of Rhinovirus/Enterovirus Test Positivity in the Inpatient and Emergency Department Settings From March 2020 to February 2021 Compared to the Same Month in Prior Years (2016-2020), United States, by Age Group [file jamanetwopen-e2254909-s001.pdf]

Supplemental Online Content

Rankin DA, Spieker AJ, Perez A, et al; NVSN Network Investigators. Circulation of rhinoviruses and/or enteroviruses in pediatric patients with acute respiratory illness before and during the COVID-19 pandemic in the US. *JAMA Netw Open*. 2023;6(2):e2254909. doi:10.1001/jamanetworkopen.2022.54909

eMethods. New Vaccine Surveillance Network Enrollment Eligibility Criteria and Research Surveillance Specimen Laboratory Testing Methods

eTable 1. Age Group-Specific Enrollment by Site and Setting, New Vaccine Surveillance Network, 2016-21

eTable 2. Absolute Number of Rhinovirus/Enterovirus Detections and Total of Children Who Received Respiratory Viral Testing From Enrollments in the Emergency Department by Surveillance Site and Age-Groups, New Vaccine Surveillance Network, 2016-2021

eTable 3. Number of Children With Rhinovirus- and/or Enterovirus ARI Study Specimens, by RT-PCR Test Type and Time Period

eFigure 1. Consort Diagram of Enrolled and Eligible Children, New Vaccine Surveillance Network, United States, November 2016–February 2021

eFigure 2. Frequency of Children Eligible and Enrolled (A) and the Absolute Number of Children With Rhinovirus/Enterovirus Detected From Research Respiratory Specimen (B), New Vaccine Surveillance Network, United States, December 2016–February 2021

eTable 4. Adjusted Odds Ratios of Rhinovirus/Enterovirus Test Positivity in the Inpatient and Emergency Department Settings From March 2020 to February 2021 Compared to the Same Month in Prior Years (2016-2020), United States

eTable 5. Adjusted Odds Ratios of Rhinovirus/Enterovirus Test Positivity in the Inpatient and Emergency Department Settings From March 2020 to February 2021 Compared to the Same Month in Prior Years (2016-2020), United States, by Age Group

This supplementary material has been provided by the authors to give readers additional information about their work.

© 2023 Rankin DA et al. *JAMA Network Open.*

eMethods. New Vaccine Surveillance Network Enrollment Eligibility Criteria and Research Respiratory Specimen Laboratory Testing Methods

Eligibility Criteria: To be eligible, children (<18 years) were required to have at least one of the following symptoms of less than 14 days duration: fever, cough, earache, nasal congestion, runny nose, sore throat, post-tussive vomiting, wheezing, shortness of breath/rapid or shallow breathing, myalgia, apnea, apparent-life threatening event, or brief resolved unexplained event.[13,15,16] Exclusion criteria were as follows: chemotherapy-associated fever and neutropenia (absolute neutrophil count $<500\times10^3/\mu L$), newborns never discharged since birth, transfer from another hospital >48 hours after admission, prior hospitalization in the last 5 days, or previous enrollment in the study in the past 14 days.[13,15,16]

Surveillance was performed at seven U.S. pediatric medical centers in the following cities: Cincinnati, OH; Houston, TX; Kansas City, MO; Nashville, TN; Pittsburgh, PA; Rochester, NY; and Seattle, WA. Year-round recruitment occurred ≥4 days per week in the ED (enrollment hours overlapping regular work and non-working times [e.g., 2:00 p.m.-10:00 p.m.]) and ≥5 days per week in the inpatient setting. For some study months, three sites restricted enrollment in the ED to children younger than 5 years (eTables 1-2 in Supplement); all available data from all sites were included in each analysis.

Data Collection & Laboratory Testing

After informed consent, the child's parent and/or legal guardian were interviewed to collect demographic information (age, sex, race, and ethnicity), symptoms, and health status using a standardized case report form. Medical chart reviews were conducted for clinical presentation, underlying medical history, admission/discharge diagnoses, and clinical outcomes. Final clinical disposition was determined by the highest level of care provided to the child during their visit (i.e., children enrolled in the ED and later hospitalized were considered an inpatient). Study respiratory specimens (e.g., mid-turbinate nasal, oropharyngeal, and/or tracheal aspirates) were collected and tested for rhinovirus/enterovirus, adenovirus, RSV, influenza, human metapneumovirus, parainfluenza virus (types 1-4), and SARS-CoV-2 (tested only in 2020–2021). For rhinovirus/enterovirus testing, some sites used platforms with combined rhinovirus/enterovirus RT-PCR outputs, and some sites used distinct rhinovirus/enterovirus RT-PCRs (which exhibit some cross-reactivity to each other, **eTable 3 in Supplement**).

Research Respiratory Specimen/Laboratory Testing Methods: Research respiratory specimens (e.g., mid-turbinate nasal, oropharyngeal, and/or tracheal aspirates) were collected at enrollment. When a research specimen was not collected, a clinically-salvaged respiratory specimen was obtained. Study specimens included both research and clinically-salvaged respiratory specimens. All study specimens were transported to each site laboratory and stored at 2°C to 8°C until processed (within 72 hours). Study specimens were tested at each site using Luminex NxTAG Respiratory Pathogen Panel (Cincinnati and Kansas City), BioFire FilmArray Respiratory Panel (Seattle), Applied Biosystems TaqMan Array Microfluidic Card (Rochester), or in-house reverse transcription-polymerase chain reaction (RT-PCR) assays (Houston, Pittsburgh, and Nashville) for rhinovirus and/or enterovirus, adenovirus, RSV, influenza, human metapneumovirus, parainfluenza virus (types 1-4), and SARS-CoV-2 (tested only in 2020–2021).[13,15,16] For testing of rhinoviruses and/or enteroviruses, two sites (Nashville and Pittsburgh) tested all study specimens using a pan-rhinovirus assay (which also detects some enteroviruses because of cross-reactivity), two sites (Rochester and Houston) used separate pan-rhinovirus and pan-enterovirus assays, and three sites (Cincinnati, Seattle, and Kansas City) used assays that provided combined rhinovirus/enterovirus outputs.[15] Each site performed

© 2023 Rankin DA et al. *JAMA Network Open.*

CDC-issued proficiency testing to ensure validity and consistency of respiratory viral detection.[13,15,16]

Statistical Analysis: Virus-specific proportions among all children with a study specimen tested and among those with a virus-positive test were evaluated by calendar months for non-enveloped respiratory viruses (1. Rhinovirus/enterovirus combined; 2. adenovirus), SARS-CoV-2, and other respiratory enveloped viruses combined (i.e., RSV, influenza, human metapneumovirus, and parainfluenza types 1–4). Rhinovirus/enterovirus RT-PCR results were combined because of cross-reactivity between the two virus groups, and because of the combined nature of the test outputs from some of the testing platforms (**eTable 3 in Supplement**).

© 2023 Rankin DA et al. *JAMA Network Open.*

eTable 1. Age Group-Specific Enrollment by Site and Setting, New Vaccine Surveillance Network, 2016-21. Inpatient enrollment included <18-year-olds year-round at all 7 surveillance sites; however, in the ED, enrollment at 3 surveillance sites was restricted to <5-year-olds for some months.

Site	Emergency department (ED) enrollment	Inpatient enrollment
Nashville, TN	<18 years, year-round	<18 years, year-round
Rochester, NY	<18 years, year-round	<18 years, year-round
Cincinnati, OH	<18 years, year-round	<18 years, year-round
Seattle, WA	<5 years, year-round<br>5-17 year-olds only enrolled during:<br>• July 1, 2017 - October 31, 2017<br>• July 1, 2018 - October 31, 2018<br>• July 1, 2019 - November 30, 2019<br>• April 1, 2020 and onwards	<18 years, year-round
Houston, TX	<18 years, year-round	<18 years, year-round
Kansas City, KS	<5 years, year-round<br>5-17 year-olds only enrolled during:<br>• July 1, 2017 - October 31, 2017<br>• July 1, 2018 - October 31, 2018<br>• July 1, 2019 and onwards	<18 years, year-round
Pittsburgh, PA	<5 years, year-round<br>5-17 year-olds only enrolled during:<br>• July 1, 2018 - October 31, 2018<br>• July 1, 2019 - November 30, 2019<br>• April 1, 2020 and onwards	<18 years, year-round

© 2023 Rankin DA et al. *JAMA Network Open.*

eTable 2. Absolute Number of Rhinovirus/Enterovirus Detections and Number of Children who Received Respiratory Viral Testing among Children Enrolled in the Emergency Department by Surveillance Site and Age-Groups, New Vaccine Surveillance Network, 2016-2021

Surveillance Site	ED, <5 years, year-round enrollment	ED, 5-17 years, year-round enrollment	ED, 5-17 years, restricted enrollment*
Nashville, TN	394/2667 (14.8%)	170/1407 (12.1%)	--
Rochester, NY	459/1471 (31.2%)	144/577 (25.0%)	--
Cincinnati, OH	483/2235 (21.6)	89/755 (11.8%)	--
Seattle, WA	250/596 (41.9%)	--	49/223 (22.0%)
Houston, TX	407/1282 (31.7%)	123/554 (22.2%)	--
Kansas City, KS	666/1414 (47.1%)	--	236/745 (31.7%)
Pittsburgh, PA	264/562 (47.0%)	--	98/264 (37.1%)

Footnote: *, in the ED, enrollment of 5-17 year-olds was limited at some sites, to the months listed in eTable 1; --, denotes not applicable, as these surveillance sites had no restricted periods.

© 2023 Rankin DA et al. *JAMA Network Open.*

eTable 3. Number Of Children with Rhinovirus- and/or Enterovirus ARI Study Respiratory Specimen, by RT-PCR Test Type and Time Period

RT-PCR type	Pre-pandemic Period***	Pandemic Period†††	Total
ARI study specimens tested across all seven sites	33,317	4,881	38,198
Any rhinovirus- and/or any enterovirus-positive specimens across all seven sites*	9,795	1,508	11,303
Positive by **either** pan-rhinovirus RT-PCR **or** by pan-enterovirus RT-PCR†	5,778	739	6,517
Positive by pan-rhinovirus RT-PCR only‡	5,536	723	3,537
Positive by pan-enterovirus RT-PCR only**	179	9	184
Positive by **both** pan-rhinovirus RT-PCR **and** by pan-enterovirus RT-PCR††	63	7	70
Positive by a platform which provides a combined rhinovirus and/or enterovirus result output‡‡	4,017	769	4,786

Footnote: Table reflects a single specimen test result for each child enrolled in our study.
* this is the summary presented in the main analysis; these data are combined from across all 7 sites.
† these data come from testing at 4 sites [Nashville, Pittsburgh, Rochester and Houston all use pan-rhinovirus RT-PCRs, and Rochester and Houston also use a pan-enterovirus RT-PCR]
‡ these data come from testing at 4 sites [Nashville, Pittsburgh, Rochester and Houston all use pan-rhinovirus RT-PCRs]
** these data come from testing at 2 sites [only Rochester and Houston use a pan-enterovirus RT-PCR]
†† these data come from testing at 2 sites [only Rochester and Houston use both a pan-rhinovirus RT-PCR and a pan-enterovirus RT-PCR]
‡‡ these data come from testing at 3 sites [Cincinnati, Kansas City and Seattle]
*** defined as 12/01/2016–03/11/2020
††† defined as 03/12/2020–02/28/2021

© 2023 Rankin DA et al. *JAMA Network Open.*

eFigure 1. Consort Diagram of Enrolled and Eligible Children, New Vaccine Surveillance Network, United States, December 2016–February 2021

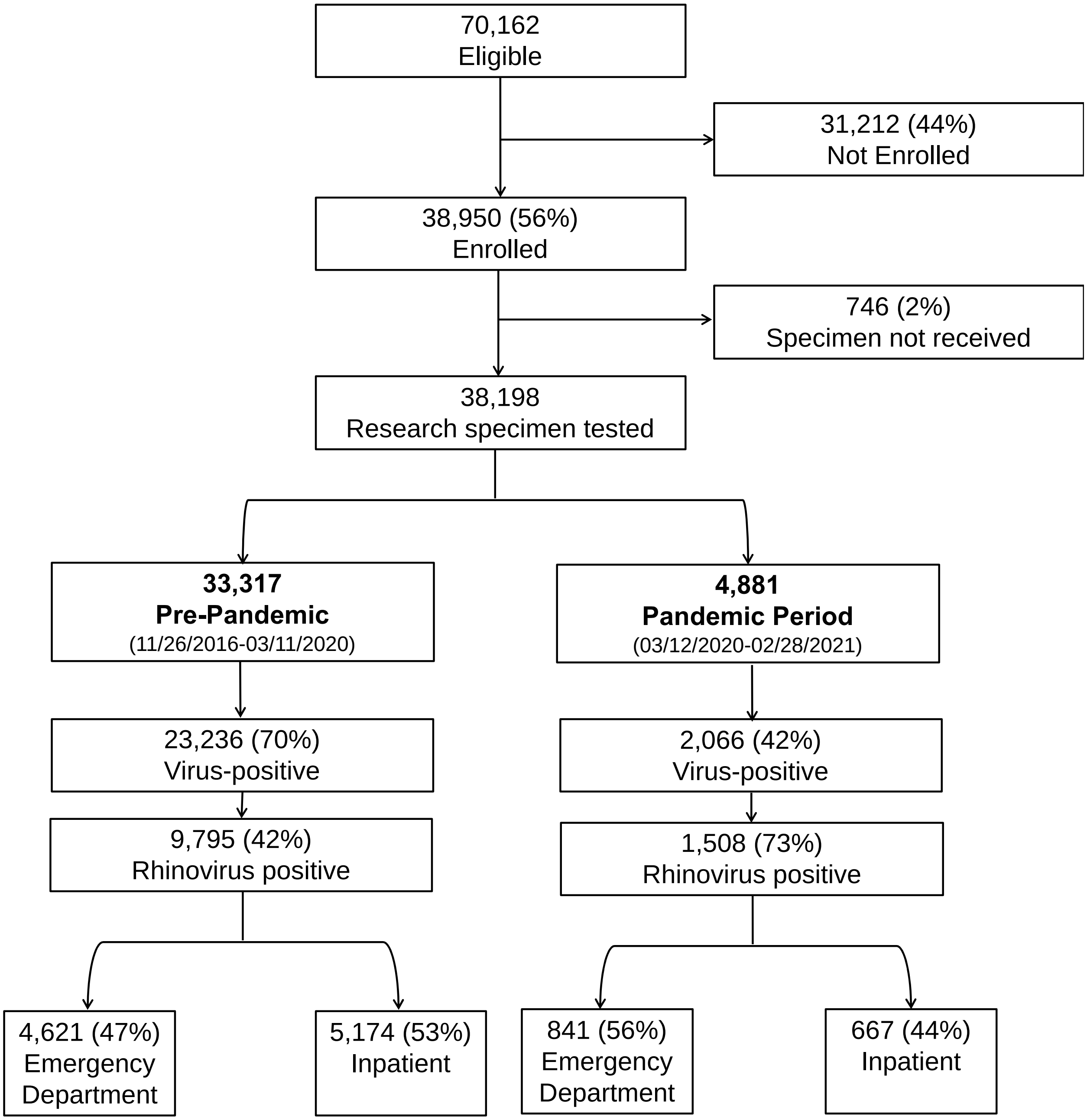

© 2023 Rankin DA et al. *JAMA Network Open.*

eFigure 2. Frequency of Children Eligible and Enrolled (A) and the Absolute Number of Children with Rhinovirus/Enterovirus Detected from Research Respiratory Specimen (B), New Vaccine Surveillance Network, United States, December 2016–February 2021
Footnote: Pandemic Period includes children eligible and enrolled into the New Vaccine Surveillance Network between March 12, 2020 to February 28, 2021

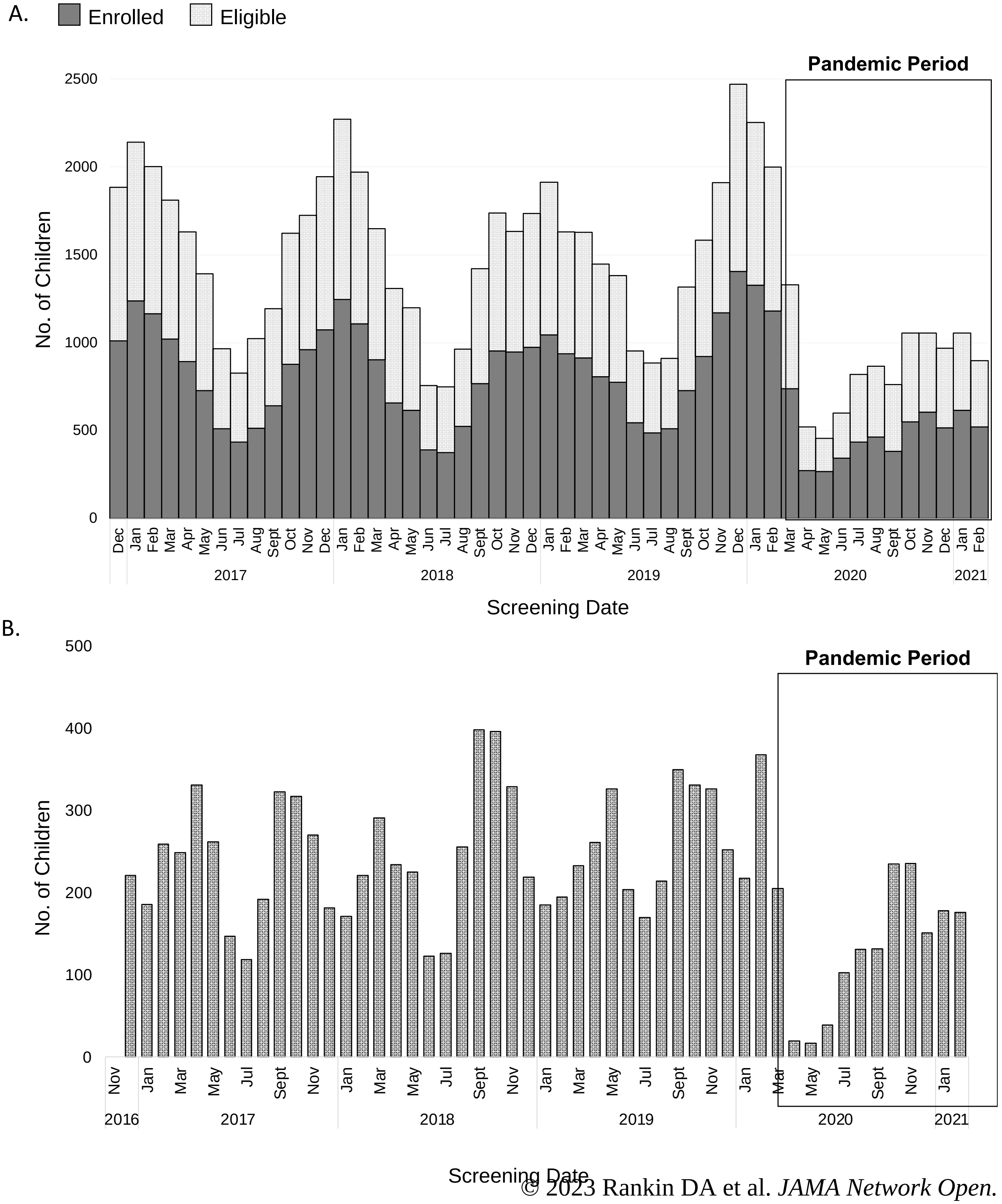

© 2023 Rankin DA et al. *JAMA Network Open.*

eTable 4. Adjusted Odds Ratios of Rhinovirus/Enterovirus Test Positivity in the Inpatient and Emergency Department Settings from March 2020 to February 2021 Compared to the Same Month in Prior Years (2016-2020), New Vaccine Surveillance Network

	Emergency Department			Inpatient		
	aOR	95% CI	p-value	aOR	95% CI	p-value
**2020**						
Mar	1.02	0.78, 1.33	0.888	1.00	0.76, 1.31	0.996
Apr	0.08	0.04, 0.19	<0.001	0.22	0.12, 0.39	<0.001
May	0.20	0.11, 0.36	<0.001	0.04	0.01, 0.11	<0.001
Jun	0.41	0.27, 0.62	<0.001	0.11	0.05, 0.23	<0.001
Jul	0.57	0.41, 0.80	0.001	0.71	0.47, 1.07	0.103
Aug	0.67	0.50, 0.90	0.008	0.41	0.28, 0.60	<0.001
Sept	0.76	0.55, 1.05	0.093	0.47	0.33, 0.67	<0.001
Oct	1.47	1.12, 1.93	0.006	1.36	1.03, 1.79	0.030
Nov	1.67	1.28, 2.19	<0.001	1.81	1.37, 2.40	<0.001
Dec	1.59	1.18, 2.16	0.003	2.37	1.74, 3.23	<0.001
**2021**						
Jan	3.01	2.30, 3.94	<0.001	2.44	1.78, 3.34	<0.001
Feb	1.68	1.26, 2.24	<0.001	1.77	1.32, 2.37	<0.001

Footnote: models were adjusted for age (continuous), sex, and insurance type (public/private/self-pay) with fixed effects for surveillance site.

© 2023 Rankin DA et al. *JAMA Network Open.*

eTable 5. Adjusted Odds Ratios of Rhinovirus/Enterovirus Test Positivity in the Inpatient and Emergency Department Settings from March 2020 to February 2021 Compared to the Same Month in Prior Years (2016-2020), New Vaccine Surveillance Network, by Age Group

	Emergency Department						Inpatient					
	<2 Years		2 to 4 years		5 to 17 years[a]		<2 years		2 to 4 years		5 to 17 years[a]	
	aOR (95% CI)	p-value	aOR (95% CI)	p-value	aOR (95% CI)	p-value	aOR (95% CI)	p-value	aOR (95% CI)	p-value	aOR (95% CI)	p-value
**2020**												
Mar	1.15 (0.79, 1.66)	0.465	0.97 (0.60, 1.59)	0.915	0.94 (0.47, 1.86)	0.850	1.00 (0.68, 1.47)	0.995	1.00 (0.57, 1.76)	0.989	0.92 (0.52, 1.64)	0.783
Apr	0.02 (0.003,0.18)	<0.001	0.21 (0.06, 0.68)	0.010	0.13 (0.03, 0.56)	0.007	0.29 (0.13, 0.68)	0.004	0.38 (0.12, 1.31)	0.127	0.12 (0.03, 0.41)	<0.001
May	0.18 (0.08, 0.39)	<0.001	0.25 (0.07, 0.92)	0.037	0.21 (0.07, 0.66)	0.007	0.07 (0.02, 0.30)	<0.001	0.05 (0.006, 0.38)	0.004	--	--
Jun	0.35 (0.20, 0.61)	<0.001	0.76 (0.29, 1.96)	0.570	0.72 (0.29, 1.79)	0.477	0.14 (0.05, 0.39)	<0.001	--	--	0.15 (0.05, 0.46)	<0.001
Jul	0.34 (0.20, 0.57)	<0.001	1.09 (0.54, 2.19)	0.818	0.80 (0.43, 1.50)	0.492	1.01 (0.52, 1.95)	0.987	0.78 (0.31, 1.94)	0.586	0.46 (0.23, 0.94)	0.032
Aug	0.65 (0.41, 1.02)	0.060	0.98 (0.53, 1.82)	0.949	0.55 (0.33, 0.91)	0.021	0.39 (0.22, 0.71)	0.002	0.50 (0.23, 1.05)	0.068	0.45 (0.23, 0.89)	0.021
Sept	0.68 (0.43, 1.09)	0.107	1.52 (0.80, 2.92)	0.204	0.53 (0.28, 1.01)	0.053	0.31 (0.17, 0.58)	<0.001	0.73 (0.38, 1.42)	0.355	0.54 (0.29, 0.99)	0.045
Oct	1.77 (1.19, 2.63)	0.005	1.38 (0.76, 2.50)	0.291	1.19 (0.70, 2.04)	0.518	1.57 (1.03, 2.39)	0.037	1.39 (0.73, 2.64)	0.319	1.34 (0.83, 2.16)	0.232
Nov	1.67 (1.12, 2.49)	0.012	1.95 (1.15, 3.31)	0.013	1.60 (0.93, 2.73)	0.087	1.83 (1.22, 2.74)	0.004	2.38 (1.30, 4.35)	0.005	1.45 (0.86, 2.44)	0.163
Dec	0.96 (0.61, 1.52)	0.873	4.28 (2.39, 7.66)	<0.001	1.46 (0.75, 2.95)	0.257	2.82 (1.82, 4.36)	<0.001	4.27 (2.11, 8.61)	<0.001	0.93 (0.50, 1.72)	0.811
**2021**												
Jan	2.71 (1.88, 3.90)	<0.001	4.93 (2.82, 8.63)	<0.001	2.84 (1.51, 5.36)	0.001	3.20 (2.08, 4.92)	<0.001	2.94 (1.45, 5.97)	0.003	1.14 (0.62, 2.13)	0.672
Feb	1.39 (0.94, 2.05)	0.096	2.70 (1.49, 4.87)	0.001	1.80 (0.95, 3.43)	0.073	2.01 (1.29, 3.12)	0.002	1.30 (0.59, 2.85)	0.513	1.91 (1.20, 3.04)	0.007

Footnote: --, indicates 0 cases were detected; [a]Inpatient enrollment included <18 year-olds year-round at all 7 surveillance sites; however, in the ED, enrollment at 3 surveillance sites was restricted to <5 year-olds for some months (see eTable 1). Age-group-specific models were used; models were adjusted for age (continuous), sex, insurance type (public/private/self-pay), with fixed effects for surveillance site.

© 2023 Rankin DA et al. *JAMA Network Open.*
