# Supplementary material for: Circulation of Rhinoviruses and/or Enteroviruses in Pediatric Patients With Acute Respiratory Illness Before and During the COVID-19 Pandemic in the US
Source: JAMA Netw Open. 2023 Feb 7;6(2):e2254909. doi: 10.1001/jamanetworkopen.2022.54909 (PMC10408278; doi:10.1001/jamanetworkopen.2022.54909)
Supplement: Supplement 2. — Nonauthor Collaborators [file jamanetwopen-e2254909-s002.pdf]

Supplemental Online Content: Nonauthor Collaborators
*First name, last name, and suffix (if applicable) are required and will appear in PubMed.

***Group Name(s): NVSN Network Investigators**							
***First Name and Middle Initial(s)**	***Last Name**	***Suffix (eg, Jr, III)**	Academic Degrees	Institution	Location (city, state/province, country)	Role or Contribution, eg, chair, principal investigator	Group (if more than 1 Group listed in the byline) and/or Subgroup (eg, Steering Committee)
Mary	Moffatt		MD	University of Missouri – Kansas C	Kansas City, MO	Sub co-investigator	
Gina	Weddle		DNP	University of Missouri – Kansas C	Kansas City, MO	Sub co-investigator	
Bonnie	Strelitz		MPH	University of Washington School c	Seattle, WA	Sub co-investigator	
Kristen	Lacombe		RN, MSN	University of Washington School c	Seattle, WA	Coordinator	
Chelsea	Rohlfs		MBA	University of Cincinnati College of	Cincinnati, OH	Coordinator	
