# Supplementary material for: Circulation of Rhinoviruses and/or Enteroviruses in Pediatric Patients With Acute Respiratory Illness Before and During the COVID-19 Pandemic in the US
Source: JAMA Netw Open. 2023 Feb 7;6(2):e2254909. doi: 10.1001/jamanetworkopen.2022.54909 (PMC10408278; doi:10.1001/jamanetworkopen.2022.54909)
Supplement: Supplement 3. — Data Sharing Statement [file jamanetwopen-e2254909-s003.pdf]

Data Sharing Statement

Rankin. Circulation of Rhinoviruses and/or Enteroviruses in Pediatric Patients With Acute Respiratory Illness Before and During the COVID-19 Pandemic in the US. *JAMA Netw Open*. Published February 07, 2023. doi:10.1001/jamanetworkopen.2022.54909

Data
Data available: Yes
Data types: Deidentified participant data
How to access data: Upon request to the corresponding author, we can provide Deidentified participant data
When available: With publication

Supporting Documents
Document types: None

Additional Information
Who can access the data: Upon request to the corresponding author
Types of analyses: for a specified purpose
Mechanisms of data availability: Upon request to the corresponding author
